# Genetic Analysis of Floral Symmetry in Van Gogh's Sunflowers Reveals Independent Recruitment of *CYCLOIDEA* Genes in the Asteraceae

**DOI:** 10.1371/journal.pgen.1002628

**Published:** 2012-03-29

**Authors:** Mark A. Chapman, Shunxue Tang, Dörthe Draeger, Savithri Nambeesan, Hunter Shaffer, Jessica G. Barb, Steven J. Knapp, John M. Burke

**Affiliations:** 1 Department of Plant Biology, University of Georgia, Athens, Georgia, United States of America; 2 Institute of Plant Breeding, Genetics, and Genomics, University of Georgia, Athens, Georgia, United States of America; The University of North Carolina at Chapel Hill, United States of America

## Abstract

The genetic basis of floral symmetry is a topic of great interest because of its effect on pollinator behavior and, consequently, plant diversification. The Asteraceae, which is the largest family of flowering plants, is an ideal system in which to study this trait, as many species within the family exhibit a compound inflorescence containing both bilaterally symmetric (i.e., zygomorphic) and radially symmetric (i.e., actinomorphic) florets. In sunflower and related species, the inflorescence is composed of a single whorl of ray florets surrounding multiple whorls of disc florets. We show that in *double-flowered* (*dbl*) sunflower mutants (in which disc florets develop bilateral symmetry), such as those captured by Vincent van Gogh in his famous nineteenth-century sunflower paintings, an insertion into the promoter region of a *CYCLOIDEA* (*CYC*)-like gene (*HaCYC2c*) that is normally expressed specifically in WT rays is instead expressed throughout the inflorescence, presumably resulting in the observed loss of actinomorphy. This same gene is mutated in two independent *tubular-rayed* (*tub*) mutants, though these mutations involve apparently recent transposon insertions, resulting in little or no expression and radialization of the normally zygomorphic ray florets. Interestingly, a phylogenetic analysis of *CYC*-like genes from across the family suggests that different paralogs of this fascinating gene family have been independently recruited to specify zygomorphy in different species within the Asteraceae.

## Author Summary

The evolution of flower shape and symmetry is of great interest to plant biologists, because it can affect pollinator behavior. Species in the flowering plant family Asteraceae exhibit flower heads that can contain both bilaterally and radially symmetric flowers. In this study, we identify a *CYCLOIDEA*-like gene that is responsible for determining flower symmetry in sunflower. Mis-expression of this gene causes a *double-flowered* phenotype, similar to those captured in Vincent van Gogh's famous nineteenth-century paintings, whereas loss of gene function causes radialization of the normally bilaterally symmetric ray florets. Interestingly, this gene is not orthologous to the *CYCLOIDEA*-like gene responsible for floral symmetry in other members of the Asteraceae, providing evidence of the parallel recruitment of different members of the same gene family for the same function.

## Introduction

The evolution of floral symmetry (i.e., the transition between actinomorphy and zygomorphy) is of great interest to plant biologists due to its apparent effect on plant-pollinator interactions and, as a consequence, rates of speciation [1]–[4]. Actinomorphy (i.e., radial symmetry) is typically considered to be the ancestral state [5], [6], with zygomorphy (i.e., bilateral symmetry) having arisen several times during the evolution of flowering plants [1], [6], [7]. Clades with zygomorphic flowers have been shown to be significantly more speciose than their sister clades with actinomorphic (i.e., radially symmetric) flowers [4], presumably because zygomorphy increases pollinator specificity, thereby setting the stage for the evolution of reproductive isolation. Given the above, it has been suggested that the evolution of zygomorphy has played an important role in plant diversification [6], [8].

**Figure 1:**
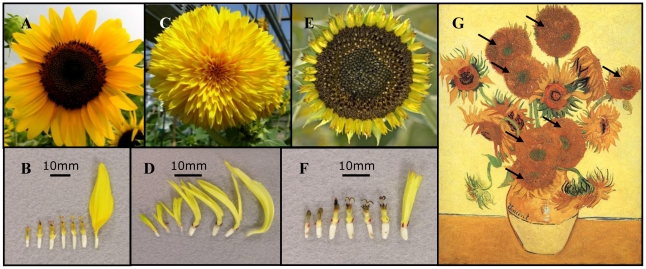
Floral symmetry in sunflower and the similarity of the double-flowered mutant to van Gogh's sunflowers. Entire inflorescences (A, C, E) and individual florets (B, D, F) from wildtype (A, B), double-flowered (C, D) and tubular (E, F) sunflower individuals. Florets are arranged left to right from the inner florets to the outer florets. (G) “Sunflowers (Still Life: Vase with Fifteen Sunflowers)” by Vincent van Gogh (1888) with double-flowered heads pointed out with arrows. Panel G was obtained from Steve Dorrington on flickr (available at http://flic.kr/p/8SsPYb) and is distributed under the terms of the Creative Commons Attribution 2.0 Generic (CC BY 2.0) License.

The Asteraceae is a particularly interesting family in which to investigate the genetics of floral symmetry. Beyond being generally recognized as the most speciose family of flowering plants [9], [10], a large number of species within this family exhibit a radiate flower head, containing both actinomorphic and zygomorphic florets within the same inflorescence. For example, in sunflower (*Helianthus annuus* L.), the wild-type (WT) inflorescence is composed of multiple whorls of actinomorphic (disc) florets surrounded by a single whorl of zygomorphic (ray) florets (Figure 1A and 1B; Figure S1). The recent elucidation of phylogenetic relationships amongst the major clades of the Asteraceae [11], [12] suggests that ray florets have evolved more than once during the diversification of this family, with a number of tribes and genera containing mixtures of radiate and discoid taxa. Ray florets have also been shown to increase pollination success in species across the family [13]–[15].

The genetic control of floral symmetry has been investigated in several species (e.g., [16], [17]). This has typically been found to involve *CYCLOIDEA* (*CYC*)-like TCP transcription factors and, at least in *Antirrhinum*, their interplay with MYB-like transcription factors [18]. Within the Asteraceae, *CYC*-like genes have since been shown to play a role in determining floral symmetry in two different species (*Gerbera* and *Senecio* [19], [20]). The *CYC*-like gene family in sunflower was previously found to be twice the size (at least ten members) of that of any other species that had been investigated to date (but see [21] for a more recent report of a similarly large number of *CYC*-like genes in the Dipsacaceae), and members of this gene family are known to have experienced positive selection and expression divergence following their duplication within the sunflower genome [22].

Sunflower mutants that show alterations in floral symmetry have been previously described (e.g., [23]–[26]), and provide an opportunity to investigate the genetic basis of this trait. For example, in *double-flowered* (*dbl*) mutants, the normally actinomorphic disc florets are elongated and vary from strongly zygomorphic towards the outside, to weakly zygomorphic towards the center of the inflorescence (Figure 1C and 1D; Figure S1), more or less reminiscent of WT ray florets. WT rays are sterile whereas WT discs are male and female fertile. In contrast some ray-like disc florets in the *dbl* mutant do not produce pollen even though anthers are present. In contrast, in *tubular-rayed* (*tub*) mutants, the normally zygomorphic ray florets are radialized and contain both stigmas and pollen-producing anthers (Figure 1E and 1F; Figure S1). Interestingly, the *dbl* mutants bear a strong resemblance to the phenotype captured in Vincent van Gogh's famous 19^th^ century sunflower paintings (Figure 1G), which have become a mainstay of van Gogh exhibits worldwide.

To further our understanding of the genetics and evolution of floral symmetry in the Asteraceae, we investigated the relationship between members of the *CYC*-like gene family and the aforementioned floral mutants in sunflower. Upon discovering that both the historically-important *dbl* phenotype as well as the *tub* phenotype are conditioned by independent mutations in the same member of the sunflower *CYC*-like gene family, we performed a phylogenetic analysis of *CYC*-like genes from across the breadth of the family. The results of this analysis suggest that different members of this fascinating gene family have been independently recruited to specify zygomorphy in species across the Asteraceae.

## Results

**Figure 2:**
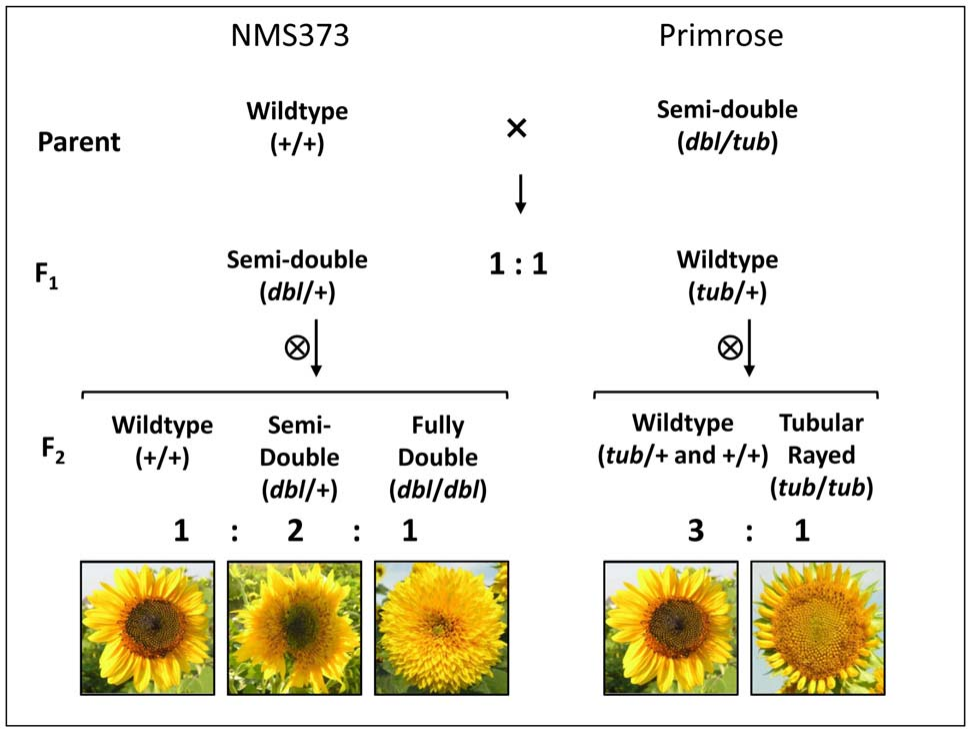
Crossing design employed to investigate the genetics of floral symmetry in sunflower with representative phenotypes shown only for the F_2_. Inferred genotypes are given in parentheses where ‘+’ indicates wild-type.

Performing controlled crosses with *double-flowered* sunflower cultivars is difficult due to reduced pollen production and difficulty in accessing the stigmas. Therefore, we initiated our investigation of floral symmetry in sunflower by crossing a ‘weak’ (i.e., intermediate) *dbl* individual (cultivar Primrose) to a WT line (cultivar NMS373) (Figure 2). F_1_ plants exhibited either weak *dbl* or WT phenotypes in a ratio not significantly different from 1∶1 (χ^2^ = 0.67, df = 1, *P* = 0.414), consistent with the effects of a single gene with codominant alleles. Three weak *dbl* F_1_ plants were selfed, and scoring of the progeny as WT, weak *dbl*, or fully *dbl* (hereafter fully *dbl* plants are referred to simply as *dbl*) revealed 1∶2∶1 (WT∶weak *dbl*∶*dbl*) segregation in all three families (all Bonferroni-adjusted *P*>0.05; Fisher's combined probability *P* = 0.09). Because modifier loci for other *dbl* lines have been reported [24], and because the phenotypic boundary between weak *dbl* and *dbl* plants is not always discrete, these families were also tested against a 3∶1 ([*dbl*+weak *dbl*]∶WT) ratio and did not differ significantly from the expectation (*P*>0.05; Fisher's combined probability *P* = 0.21).

Self-pollination of four of the WT F_1_ plants revealed a novel phenotype (*tub*; tubular-ray florets; Figure 1E and 1F) amongst the resulting progeny. In this case, the phenotypic ratios were not significantly different from 3∶1 (WT∶*tub*) (all *P*>0.05; Fisher's combined probability *P* = 0.10), consistent with the effects of a single gene with a recessive, mutant allele. Selfing of the *tub* plants resulted in only *tub* offspring. Moreover, no double-flowered plants with tubular ray florets were observed in any of the F_2_ families, suggesting that the *dbl* and *tub* phenotypes are allelic, or due to the effects of tightly linked genes. This view is supported by the observation that crosses between *dbl* and *tub* plants resulted in weak *dbl* offspring, and that self-pollination of two of these individuals resulted in a 3∶1 ([*dbl*+weak *dbl*]∶*tub*) segregation ratio (both *P*>0.05). The weak *dbl* phenotype therefore appears to correspond to *dbl*/WT (or *dbl*/*tub*) heterozygotes.

Genetic mapping in these Primrose×NMS373 F_2_ families (see Materials and Methods) revealed that both traits map to the same region of sunflower linkage group nine, coincident with the position of three *CYCLOIDEA*-like (*CYC*) genes mapped in an earlier study (*HaCYC2b*, *HaCYC2c*, and *HaCYC2e*; [22]). One of the three *CYC*-like genes (*HaCYC2c*) showed sequence polymorphism in this population, and exhibited complete cosegregation with the *tub* phenotype in two WT∶*tub* populations. All three genes showed sequence polymorphism in a second Primrose×WT population (WT cultivar Moulin Rouge) and, upon mapping, were shown to cosegregate with each other and with the *dbl* phenotype.

Because of their role in determining floral symmetry in other species, these *CYC*-like genes are good candidates for being involved in specifying the zygomorphy of WT ray florets. Previous analyses demonstrated that all three *CYC*-like genes are expressed in floral tissues [22]; however, one of them (*HaCYC2c*) exhibits ray-specific expression while the other two (*HaCYC2b* and *HaCYC2e*) are expressed across multiple floral tissues including rays, discs, ovules and stigmas [22].

**Figure 3:**
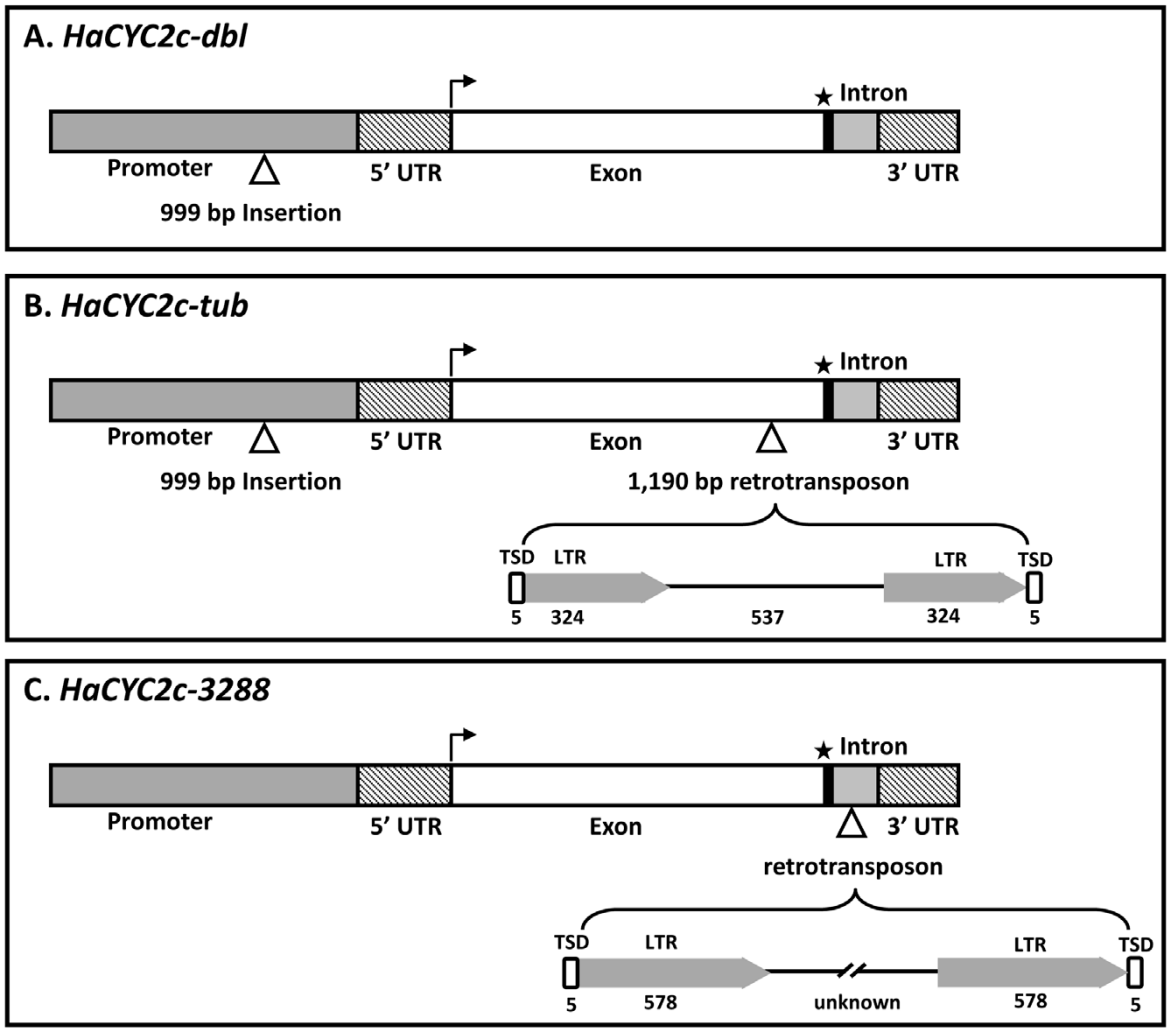
Schematic diagram of the mutant alleles of *HaCYC2c*. The bent arrow and star indicate the start and stop codons respectively. Insertions are indicated by open triangles.

Sequencing from the WT parent (NMS373), as well as true-breeding *dbl* and *tub* lines, revealed that *HaCYC2b* and *HaCYC2e* have identical, uninterrupted coding sequences in all three types, suggesting that these genes are not responsible for the observed phenotypes. In contrast, the sequences of *HaCYC2c* from both the *dbl* and *tub* lines (alleles *HaCYC2c-dbl* and *HaCYC2c-tub*, respectively) contained a 999 bp insertion upstream of the start codon, and the *HaCYC2c-tub* allele contained an additional 1190 bp insertion in the coding region (Figure 3).

**Figure 4:**
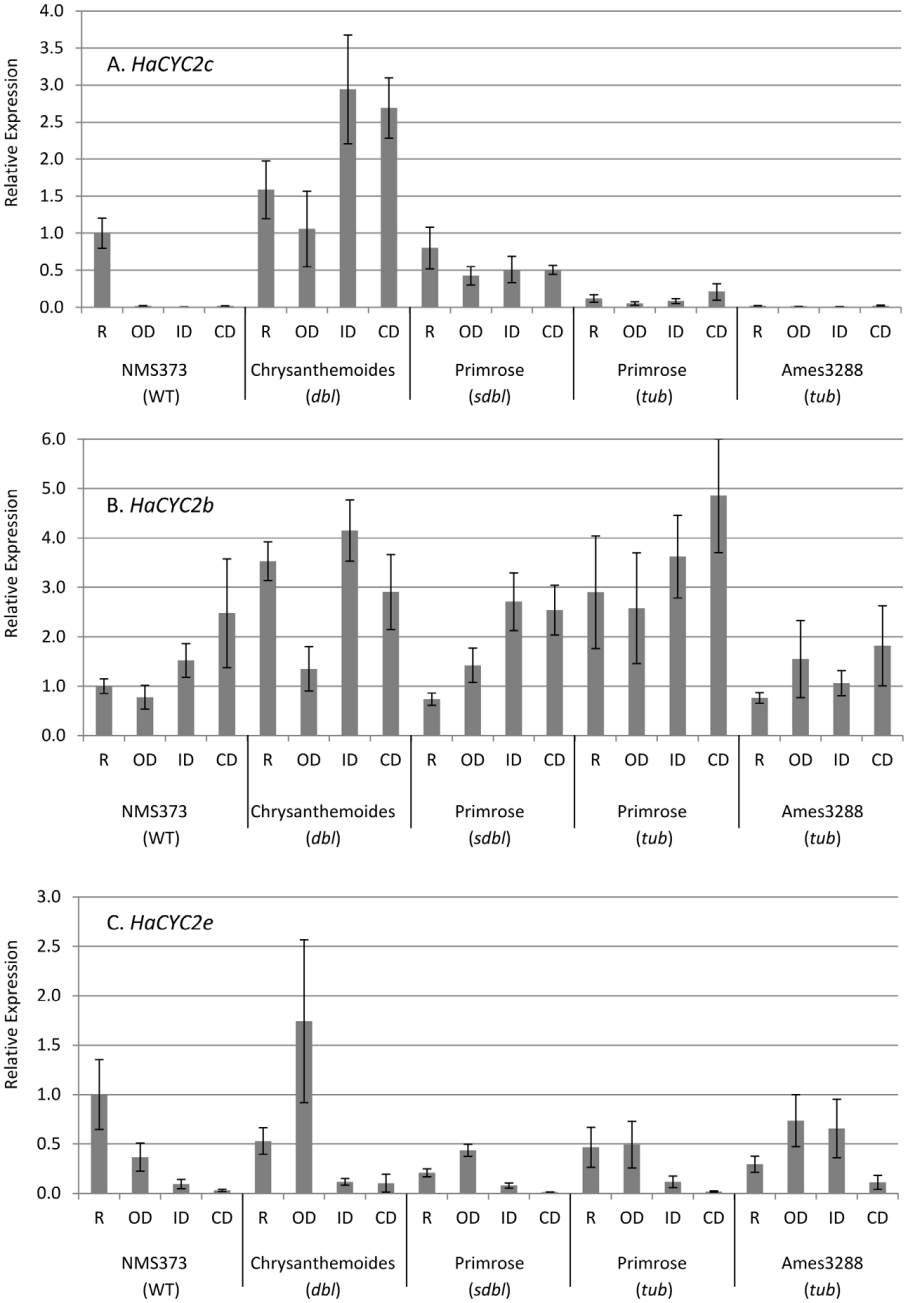
Quantitative RT–PCR results for the expression analysis of *HaCYC2c* in sunflower. Tissues are ray florets (R), outer discs (OD), intermediate discs (ID) and central discs (CD) and the sunflower lines, with phenotypes in parentheses, are given under the graph. Values were normalized to actin, and are graphically scaled to NMS373 (i.e., WT) ray florets, with error bars depicting the standard error of four biological replicates.

The 999 bp upstream insertion showed no hallmarks of being a transposon or other mobile DNA element. In contrast, inspection of the sequence of the 1190 bp insertion in *HaCYC2c-tub* revealed a 5 bp target-site duplication (TSD), the presence of identical 324 bp long terminal repeats (LTRs), a primer binding site, and a polypurine tract, suggesting that this insertion is a terminal repeat retrotransposon in miniature (TRIM) [27]. Both mutations in *HaCYC2c* affect gene expression, causing a deviation from the WT ray-specific expression of *HaCYC2c*, as follows. In the *dbl* flower head, *HaCYC2c* is expressed in all florets across the inflorescence (i.e., in both disc and ray florets), whereas reduced *HaCYC2c* expression was detected across the head in *tub* mutants (Figure 4A). It thus appears that the 999 bp insertion affects a ‘ray-floret-specific’ element in the promoter region of *HaCYC2c*, as evidenced by the expression across all floret types in both the *dbl* and *tub* lines. The TRIM insertion apparently reduces expression of *HaCYC2c* in *tub* mutants, and also results in the production of a premature stop codon, presumably preventing its WT function. In contrast, expression patterns for *HaCYC2b* were generally similar across genotypes (Figure 4B) and, while *HaCYC2e* showed some expression variation across genotypes (Figure 4C), there was no clear evidence of disrupted gene expression resulting in the observed mutant phenotypes. For example, inner and central discs (ID and CD) showed low expression (similar to WT) in *dbl* and *sdbl* mutants despite their ray-like appearance, and the Primrose and Ames3288 *tub* mutants (see below for details on Ames3288) did not show consistent changes relative to WT. These findings suggest that *CYC2c* is required for zygomorphy in normal WT ray florets, with ectopic expression in disc florets (i.e., *HaCYC2c-dbl*) causing them to become zygomorphic in *dbl* mutants, and greatly reduced expression coupled with a truncated mRNA (i.e., *HaCYC2c-tub*) causing a loss of zygomorphy in *tub* mutants.

To further investigate this possibility, *HaCYC2b*, *2c*, and *2e* were sequenced from a second line with tubular ray florets (USDA accession Ames3288). Whilst *HaCYC2b* and *2e* sequences were identical between Ames3288 and the Primrose *tub* mutant line (and WT), *HaCYC2c* contained a unique mutation (*HaCYC2c-3288*; Figure 3C). In this second tubular-rayed line, a putative retrotransposon (identified on the basis of a 5 bp TSD and 578 bp identical LTRs) had inserted 55 bp downstream of the stop codon, interrupting the intron in the 3′ untranslated region (UTR), and resulting in an almost complete loss of expression (Figure 4A; see below for additional discussion). When this line was crossed with individuals homozygous for the Primrose-derived *HaCYC2c-tub* mutant allele, all resulting offspring (at least five from each of four independent crosses) exhibited tubular rays (i.e., there was no complementation), presumably due to non-complementary knock-out mutations at *HaCYC2c*. We therefore conclude that the mutations are indeed allelic, and that mutations in the *HaCYC2c* gene are responsible for the *dbl* and *tub* phenotypes.

*HaCYC2c* was also sequenced from three other double-flowered sunflower lines. Two of these, Sungold Tall and *Chrysanthemoides* [24], both harbor the *HaCYC2c-dbl* allele with the upstream insertion. The third, Teddy-bear, was found to be heterozygous for the same alleles that were present in the original cv. Primrose individual (i.e., *HaCYC2c-dbl* and *HaCYC2c-tub*). When additional Teddy-bear plants were grown and self-pollinated, we observed some individuals with the *dbl* phenotype and others with tubular ray florets. Hence, the *HaCYC2c-dbl* and *HaCYC2c-tub* alleles appear to be segregating in this line in the same manner as in Primrose. Expression of *HaCYC2c* in *Chrysanthemoides* follows the same overall pattern as *Primrose-dbl*, confirming our observation of mis-expression of this mutant allele in a different genetic background (Figure 4A).

In order to further examine whether the three insertions (*HaCYC2c-dbl*, *HaCYC2c-3288* and *HaCYC2c-tub*) are responsible for the mutant phenotypes, we carried out a polymerase chain reaction (PCR) screen of a diverse collection of 108 sunflower lines that exhibit WT floral morphology (see Materials and Methods). This screen revealed that all three insertions are indeed unique to the respective mutant lines (i.e., they were never observed in WT lines), providing further evidence that the *HaCYC2c* gene plays a critical role in proper floret development, with mis-expression and loss of function of this gene giving rise to the *dbl* and *tub* phenotypes, respectively.

**Figure 5:**
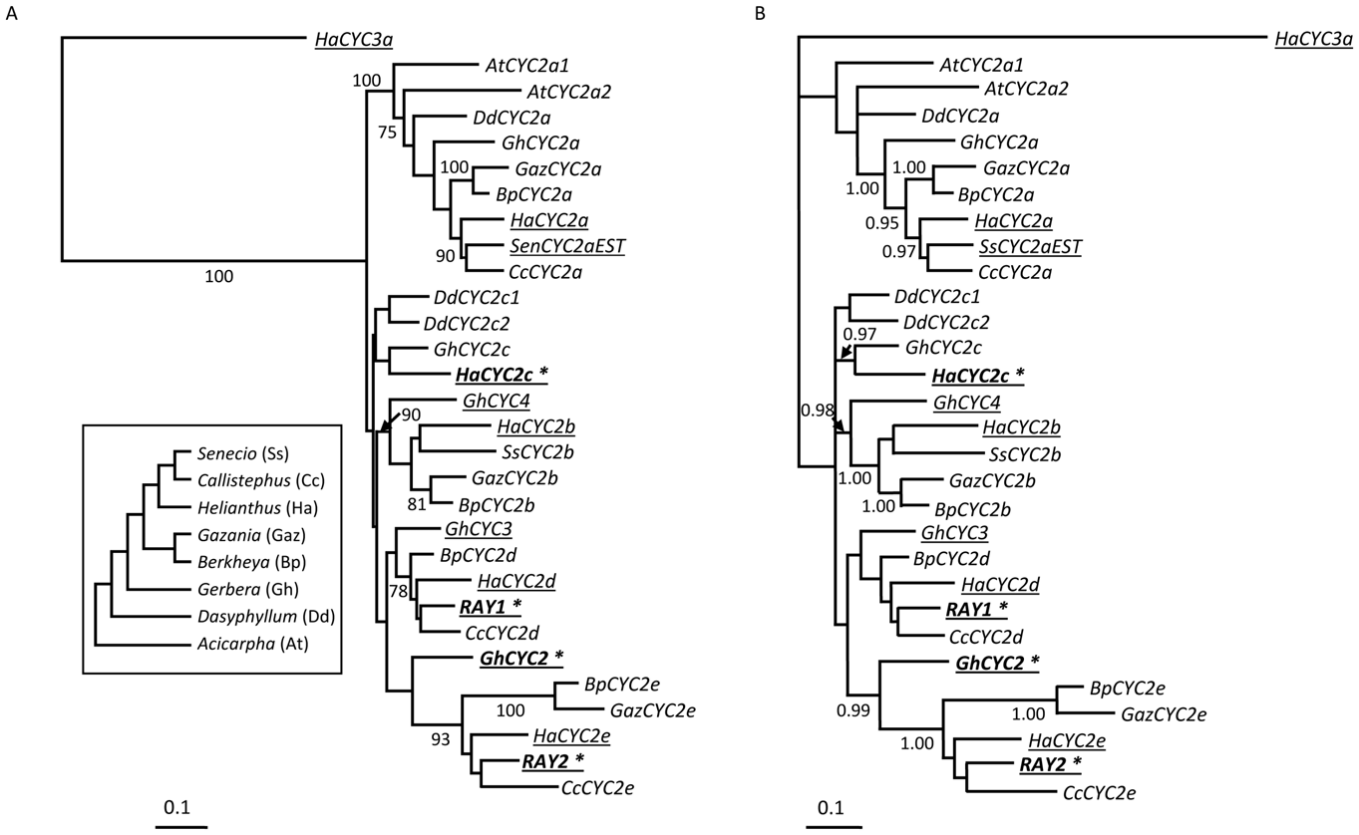
Maximum likelihood and Bayesian gene trees showing relationships between *CYCLOIDEA* gene sequences from the Asteraceae. (a) Maximum likelihood, and (b) Bayesian gene trees. Nucleotide sequences of the conserved TCP and R domains and the intervening sequence were used in the phylogenetic reconstructions. Inset depicts the relationships between the species investigated (according to Panero and Funk, 2008). *CYC2* sequences were either PCR amplified from members of the Asteraceae (see Materials and Methods) or taken from Genbank (underlined). Previously published sequences are named according to the original publications. For genes isolated herein, species names are abbreviated as follows: *Acicarpha* (At), *Berkheya* (Bp), *Callistephus* (Cc), *Dasyphyllum* (Dd), *Gazania* (Gaz), *Gerbera* (Gh), *Helianthus* (Ha), and *Senecio* (Ss) and are named according to the subgroup (a, b, c, d, or e) in which they fall based on the sunflower paralog names. Loci playing a role in ray floret symmetry (refs [19], [20]; this study) are indicated in bold and denoted with an asterisk. Bootstrap values (a: 100 replicates) are shown where greater than 70%, and Posterior Probabilities (b) where greater than 0.95.

To better understand the diversification of the *CYC*-like gene family within the Asteraceae, *CYC2*-like genes were isolated from other radiate members of the family, a basal species with actinomorphic and zygomorphic florets and a member of the sister family, the Calyceraceae, with only actinomorphic flowers (see Materials and Methods), and gene trees were constructed (Figure 5). The focus here was on the *CYC2*-like subfamily because it is *CYC2* genes (as opposed to *CYC1* or *CYC3*) that are responsible for specifying zygomorphy in a wide range of species [3], [16]–[20]. The gene trees suggest that the sunflower *HaCYC2c* gene, which is responsible for specifying ray floret formation, is paralogous to (i.e., not the direct ortholog of) the *Senecio* and *Gerbera CYC*-like genes that have previously been shown to influence floral symmetry in these other members of the Asteraceae (Figure 5). The *Gerbera* locus controlling floral symmetry is *GhCYC2*, which is not orthologous to *HaCYC2c*; rather, this gene is part of the *HaCYC2e*-like clade, along with one of the two *Senecio* floral symmetry genes, *RAY2*. Although there is only weak support for some branches in these trees, the second *Senecio* symmetry gene, *RAY1*, also falls outside of the well-supported *HaCYC2c* clade, grouping instead with *HaCYC2d*.

## Discussion

The evolution of floral zygomorphy has been proposed as a key innovation in angiosperm evolution, with elevated divergence rates thought to be the result of adaptation to specialized pollinators [1], [2], [28]. As noted above, zygomorphy has evolved on multiple occasions, and the role of *CYCLOIDEA*-like genes in specifying zygomorphy has been implicated in a number of these cases [3], [16]–[20]. The connection between *CYC*-like genes, floral symmetry, and pollination syndromes is further evidenced by instances in which changes in *CYC* gene number and/or expression patterns correlate with alterations of floral symmetry [29]–[34], and at least one case in which the loss of *CYC*-like genes (and their downstream targets) caused a reversion from zygomorphic to actinomorphic flowers and a concomitant change in pollination syndrome from insect to wind pollination [35].

In sunflower, which has both actinomorphic and zygomorphic florets within the same inflorescence (Figure 1), our results indicate that *HaCYC2c* is necessary for floral zygomorphy, with individuals that are homozygous for a loss-of-function mutation (either due to a premature stop codon or to a reduction/loss of expression of *HaCYC2c*) exhibiting actinomorphic ray florets. Moreover, the historically important *double-flowered* mutation, which was captured by van Gogh in his famous late-19^th^ century sunflower paintings, appears to be conditioned by a different mutation in the same gene. In this case, ectopic expression of *HaCYC2c* across the entire inflorescence results in the transformation of normally actinomorphic disc florets into zygomorphic ray-like florets.

It has previously been shown that *CYCLOIDEA*-like genes that control zygomorphy act in a dorsal-specific manner, repressing cell growth in this region and allowing outgrowth of the ventral petals [16], [17]. The phenotypes of the *tub* and *dbl* mutants are therefore in line with this mode of gene action. That is, loss of function (*tub*) causes radialization of the ray florets, whereas expression in disc florets (*dbl*) results in ventralization. It is interesting that, in the *tub* lines, the outer florets were not simply radialized ray florets; rather, they were effectively transformed into elongated disc florets, as indicated by the presence of stigmas and pollen-producing anthers. This result, along with the observed transformation of disc florets in *dbl* mutants, suggests that *HaCYC2c* is responsible for specifying ray vs. disc floret identity, and not simply required for zygomorphy of the ray florets – though the situation is clearly complex, as the radialized ray florets still exhibit elongation and the zygomorphic disc florets in *dbl* mutants still produce anthers and stigmas.

Given that all four of the *dbl* lines investigated herein carried the same mutant allele (*HaCYC2-dbl*), it seems likely that this mutation arose just once, and has been incorporated into multiple cultivars because it produces a desirable floral morphology. In contrast, sunflower lines that exhibit tubular ray florets appear to have arisen at least twice. In one case (*HaCYC2c-tub*), a transposon insertion into the coding region of this gene appears to have resulted in reduced expression and production of a premature stop codon, resulting in radialization of the ray florets. The second case (*HaCYC2c-3288*) involves the insertion of a different transposon into an intron in the 3′ UTR of this gene, resulting in a loss of gene expression and radialization of the ray florets. In fact, previous studies have revealed that introns in UTRs can play a vital role in gene regulation [36], [37]; hence, the interruption of an intron in a UTR has the potential to disrupt gene expression, as appears to be the case here.

It is noteworthy that the *HaCYC2c*-*dbl* and *HaCYC2c-tub* alleles both contain the 999 bp upstream insertion resulting in a loss of ray-specific expression, and differ only by the presence (*HaCYC2c-tub*) or absence (*HaCYC2c*-*dbl*) of the TRIM insertion into the coding region (Figure 3). This finding implies that the *HaCYC2c-tub* allele is derived from *HaCYC2c-dbl*, and further suggests that the family of elements to which the *CYC2c* TRIM insertion belongs may still be active in the sunflower genome. The apparent recency of this insertion is further evidenced by the fact that the element in question appears to be intact, complete with identical LTR sequences. In contrast, the *HaCYC2c-3288* allele appears to be derived from a WT *HaCYC2c* allele. The high level of sequence similarity between the coding regions of WT alleles and *HaCYC2c-3288*, along with the identical LTR sequences, likewise suggest that this was an evolutionarily recent insertion event.

We previously suggested [22] that the large number of *CYC*-like genes in sunflower was due in part to a whole genome duplication event at the base of the Heliantheae (i.e., the sunflower subfamily) [38]. These duplications are, however, clearly shared with other members of the family outside the Heliantheae, suggesting that the radiation of this gene family occurred earlier in the evolution of the Asteraceae. Moreover, the apparent absence of *CYC2b, c, d*, and *e* genes from *Acicarpha* and *CYC2b, d*, and *e* genes from *Dasyphyllum* (Figure 5) suggests that some members of this gene family may have been lost in certain lineages, or that some duplications occurred since the split between *Gerbera* and *Dasyphyllum*, giving rise to the *2b, d, e* clade. A more thorough investigation of the genetics of floral symmetry in the basal members of the family is clearly warranted.

Interestingly, inspection of the *CYC2*-like gene tree from across the Asteraceae (Figure 5) reveals that the sunflower *HaCYC2c* gene is not the direct ortholog of the *CYC2* genes that have been shown to be responsible for specifying zygomorphy in two other members of the Asteraceae (i.e., *Senecio* and *Gerbera*). Rather, *HaCYC2c* appears to be paralogous to these genes, suggesting that the evolution of zygomorphy within the Asteraceae, which is thought to have occurred multiple times [11], [39], involved the parallel co-option of different members of the same gene family for an analogous function.

It is possible that other, closely related *CYC*-like genes have functions similar to *HaCYC2c*. In this context, the role of *HaCYC2e* is of particular interest because: (1) *HaCYC2e* and *HaCYC2c* both co-segregate with the mutant phenotypes, and (2) *HaCYC2e* appears to be the true sunflower ortholog of *RAY2* and *GhCYC2* (Figure 5b), which are known to influence zygomorphy in *Senecio* and *Gerbera*. In this light, it is noteworthy that *HaCYC2e* is much more broadly expressed than *HaCYC2c* in the WT inflorescence ([22] and Figure 4; i.e., the former is expressed in all floral tissues examined, albeit at low levels near the center of the disc, while the latter is ray-specific), and that *HaCYC2e* expression patterns did not clearly correlate with the mutant phenotypes. Moreover, the upstream and coding sequences of *HaCYC2e* were identical between WT, *dbl*, and *tub* individuals, whereas for *HaCYC2c*, mutations that gave rise to altered expression patterns and/or predicted protein sequences were observed. Nonetheless, a better understanding of the possible role of *HaCYC2e* in floral development in sunflower awaits further investigation. We can, however, conclude that *HaCYC2c* is a key transcription factor in the developmental pathway resulting in the development of zygomorphic ray florets in sunflower.

The parallel evolution of adaptive traits has been documented many times [40]. In some cases, such parallel phenotypic changes have been shown to result from parallel molecular changes [41], [42]. In other cases, however, it has been shown that parallel phenotypic evolution results from the evolution of different genes to perform the same function, suggesting that independent gene co-option may be an important mechanism for the origin of evolutionary novelty [43]–[46]. In contrast to the co-option of unrelated genes/proteins to fill the same functional role in independent evolutionary lineages, the co-option of different, but related genes (i.e., those with ancestral similarity, such as different members of the same gene family) has been less well documented. It has, however, recently been shown that O_2_-transporting hemoglobins have evolved independently from different members of the globin gene superfamily in jawed vs. jawless vertebrates [47]. While *CYC2*-like genes have been implicated in controlling zygomorphy in several plant species (reviewed in [48]), our work provides evidence that subfunctionalization of different paralogs within this gene family has resulted in the independent evolution of an analogous adaptive trait in evolutionarily-independent lineages.

## Materials and Methods

### Plant Material and Crosses

Most of the sunflower (*Helianthus annuus* L.) lines used in this research were obtained from the USDA North Central Regional Plant Introduction Station (NCRPIS; Ames, IA). The exceptions were *Chrysanthemoides* [24], which was kindly provided by Dr. Claudio Pugliesi (Università di Pisa, Italy) and Moulin Rouge, which was obtained from Johnny's Selected Seeds (Winslow, Maine, USA). USDA Plant Introduction (PI) numbers for the wild-type parent and mutant lines are as follows: NMS373 (PI 597362), Primrose (PI 490320), Ames3288 (PI 650394), Teddy-bear (PI 650838), Sungold Tall (PI 490322). The 108 WT lines that were screened for insertions in *HaCYC2c* (see below) are listed in Table S1. When crosses were made, florets were emasculated and heads were bagged pre- and post-pollination to prevent pollen contamination. Individuals were scored as wild-type (WT), weak double-flowered (weak *dbl*), fully double-flowered (*dbl*), or tubular-rayed (*tub*) based on the morphology of their florets.

### Genetic Mapping

Microsatellite markers showing linkage with the three phenotypes (i.e., WT vs. *dbl*; WT vs. *tub*) were first identified by extracting DNA from individuals of the three phenotypes using MagAttract 96 or DNeasy DNA extraction kits (Qiagen, Valencia, CA, USA), bulking equal amounts of DNA from ten individuals per phenotype (WT, *dbl*, and *tub*) and genotyping the bulked samples using multiplexed polymerase chain reaction (PCR) amplification [49]. Alleles were visualized using GeneMapper (Applied Biosystems, Carlsbad, California, USA). Both phenotypes showed an association with marker ORS844 on linkage group (LG) nine, so additional markers (ORS1265, ZVG39, CRT250, and ORS176; also from ref. 49) from this LG were genotyped in 96 F_2_ progeny from a WT×*dbl* cross and 192 F_2_ progeny from two WT×*tub* crosses, all of which were derived from the original NMS 373×Primrose cross (Figure 2). Genetic mapping was carried out using Mapmaker 3.0 (Lander et al. 1987; Lincoln et al. 1992) following ref. 22. The *HaCYC2c-tub* insertion was also mapped in the two Primrose×NMS373 F_2_ families segregating for WT∶*tub* phenotypes (n = 192 F_2_ plants) and complete co-segregation between the *HaCYC2c-tub* allele and the *tub* phenotype was observed.

Because the alleles of both *HaCYC2b* and *HaCYC2e* were identical in the parents of the aforementioned mapping populations, these genes could not be re-mapped in these crosses. Therefore, single nucleotide polymorphism (SNP) markers were developed for all three loci in an independent WT (Moulin Rouge)×Primrose mapping population (n = 195 F_2_ plants) where they showed sequence differences. Loci were PCR amplified in 5 µl reaction volumes containing 2 ng genomic DNA, 1 µL 1× LightCycler 480 Genotyping Master mix (Roche Diagnostics, Indianapolis, IN, USA), 1.0 µM of excess primer, 0.5 µM of limiting primer, and 0.2 µM of both the sensor and anchor probes (Table S2). PCR was performed in the Light Cycler 480 (Roche) for 45 cycles with 10 sec 95°C, 15 sec at 55°C, and 20 sec at 72°C. The final melting cycle was performed by raising the temperature to 95°C for 3 min, lowering the temperature to 40°C for 3 min, and increasing the temperature to 85°C with continuous fluorescent acquisition 5 times/degree. The fluorescence signal (F) was plotted in real time against temperature (T) to produce melting curves for each sample, and the melting curves were converted to negative derivative curves of fluorescence with respect to temperature (-dF/dT) by the LightCycler Data Analysis software (Roche).

### DNA Sequencing and Allele Screening

*HaCYC2*-like genes were PCR-amplified (see primer sequences in Table S2) and sequenced using previously established protocols (e.g., ref. 50). Insertions in *CYC2c* were found in the mutant genotypes that were initially screened (Primrose and Ames3288; Genbank accession numbers HQ891026–HQ891029). *HaCYC2c* was also sequenced from three additional *dbl* mutants (Sungold Tall, *Chrysanthemoides*, and Teddy-bear; Genbank accession numbers JF489909–JF489913). Primer pairs (Table S2) specific to all three insertions (for each insertion, one primer was placed inside the insertion, with the other being placed in the gene itself) were then used to PCR amplify and screen a diverse panel of 108 WT cultivated sunflower lines [51] for presence or absence of each insertion. DNA extraction was carried out as above and PCR carried out as previously described [50]. Amplicons were visualized via agarose gel electrophoresis and staining with ethidium bromide. Presence or absence of a PCR product indicated presence/absence of an insertion. A positive control PCR was carried out for each individual using another pair of primers specific to *HaCYC2c* to protect against the possibility of false negatives.

### Expression Analyses

Gene expression analyses were carried out using quantitative reverse-transcriptase (qRT)-PCR on RNA isolated from WT (NMS373), *dbl*, *tub*, Ames3288 and *Chrysanthemoides* individuals. Briefly, petal tissue was collected for each genotype/floret type combination at a stage when the sunflower head was fully open. Tissues were collected from four different floret types, namely: ray florets, outer discs, intermediate discs (halfway between the outer and central florets), and central disc florets. RNA was extracted using the guanidium isothiocyanate method followed by isolation using the RNeasy Mini Kit (Qiagen, Valencia, CA) using previously established protocols [22]. Synthesis of cDNA was performed using 500 ng of total RNA after removal of genomic DNA using Qiagen on-column DNase treatment. ImProm II reverse transcriptase (Promega Corporation, WI, USA) and oligo dT (15) primers were used to perform reverse transcription in a 30 µl reaction volume. The cDNA was diluted with 80 µl of water for all gene expression analyses. All the qRT-PCR analyses were performed using an Eppendorf realplex^2^ real-time PCR system (Eppendorf, Hauppauge, NY) with primers in Table S2. The reaction conditions were as follows: 50°C for 2 min; 95°C for 10 min; 40 cycles of 95°C (15 s), 59°C (20 s) and 68°C (30 s). Melt-curve analyses were performed after the PCR. A single distinct peak was observed for both the target (*HaCYC2b*, *2c*, and *2e*) and control (actin) genes indicating the specific amplification of a single product. Relative expression (scaled against WT ray florets) was calculated using the Pfaffl method [52].

### Gene Discovery and Phylogenetic Analysis

Degenerate PCR primers were employed as previously described [22] to amplify the central portion (i.e., the region between the TCP and R domains) of the *CYC*-like genes from other members of the Asteraceae (*Berkheya purpurea* [subfamily Cichorioideae], *Callistephus chinensis* [Asteroideae], *Dasyphyllum diacanthoides* [Barnadesioideae], *Gazania hybrida* [Cichorioideae], *Gerbera hybrida* cultivar [Mutisioideae], *Senecio squalidus* [Asteroideae]) as well as *Acicarpha tribuloides* (Calyceraceae; outgroup). Seeds of most of these species were obtained from Chileflora (www.chileflora.com), Chiltern Seeds (www.chilternseeds.co.uk), or the USDA (see above). The exceptions were *Senecio* (seed from Richard Abbott, University of St. Andrews) and *Acicarpha* (leaf material from Leigh Johnson, Brigham Young University). PCR, cloning, and sequencing were carried out as before [50]. These species were chosen to represent a broad cross-section of the family. However, we focused on radiate species where possible. The exceptions are *Dasyphyllum*, with an inflorescence made up of actinomorphic disc and zygomorphic bilabiate florets, and *Acicarpha*, which approximates an inflorescence with only actinomorphic flowers.

The *CYC*-like gene family in the Asteraceae is made up of three subfamilies, and the focus here was on *CYC2*-like genes. Therefore, *CYC* clones from the above reactions that showed similarity to *CYC2* genes from *Helianthus* were further characterized via genome walking (as described previously [22]) to obtain the entire TCP and R domains. A nucleotide alignment was next obtained for the conserved TCP and R domains as well as the intervening region using ClustalW2 [53] followed by manual adjustment such that indels were in multiples of three. Maximum likelihood analysis was then carried out using PhyML [54] with 100 bootstrap replicates and Mr. Bayes [55] to produce gene trees.

### Accession Numbers

Sequence data have been deposited into the Genbank DNA database (www.ncbi.nlm.nih.gov/genbank) under accession numbers HQ891026–HQ891029 and JF489909–JF489913 (*HaCYC2c*), JF489906–JF489908, JQ594983 (*HaCYC2b*), JF489914–JF489916, JQ594982 (*HaCYC2e*), and JF299240–JF299257 (Asteraceae *CYC2* sequences).

## Supporting Information

Figure S1 Floret morphology in the WT mutant (Primrose *dbl* and Primrose *tub*) lines. Ovaries and stigmas have been removed from the florets, which have been split laterally so that the anthers can be seen. Scale bars (10 mm) are indicated in white. In each panel outer florets are on the left, and the innermost florets on the right.
(PDF)

Table S1 108 diverse sunflower lines used in the PCR assay for insertions in *Hacyc2c*. All lines are available from the USDA (http://www.ars-grin.gov/npgs/index.html) with the exception of SF33 and SF230 available from the French National Institute for Agricultural Research (INRA).
(PDF)

Table S2 Primer sequences used throughout this study.
(PDF)
