# Supplementary figures and images for: Genetic Analysis of Floral Symmetry in Van Gogh's Sunflowers Reveals Independent Recruitment of CYCLOIDEA Genes in the Asteraceae
Source: PLoS Genet. 2012 Mar 29;8(3):e1002628. doi: 10.1371/journal.pgen.1002628 (PMC3315478; doi:10.1371/journal.pgen.1002628)

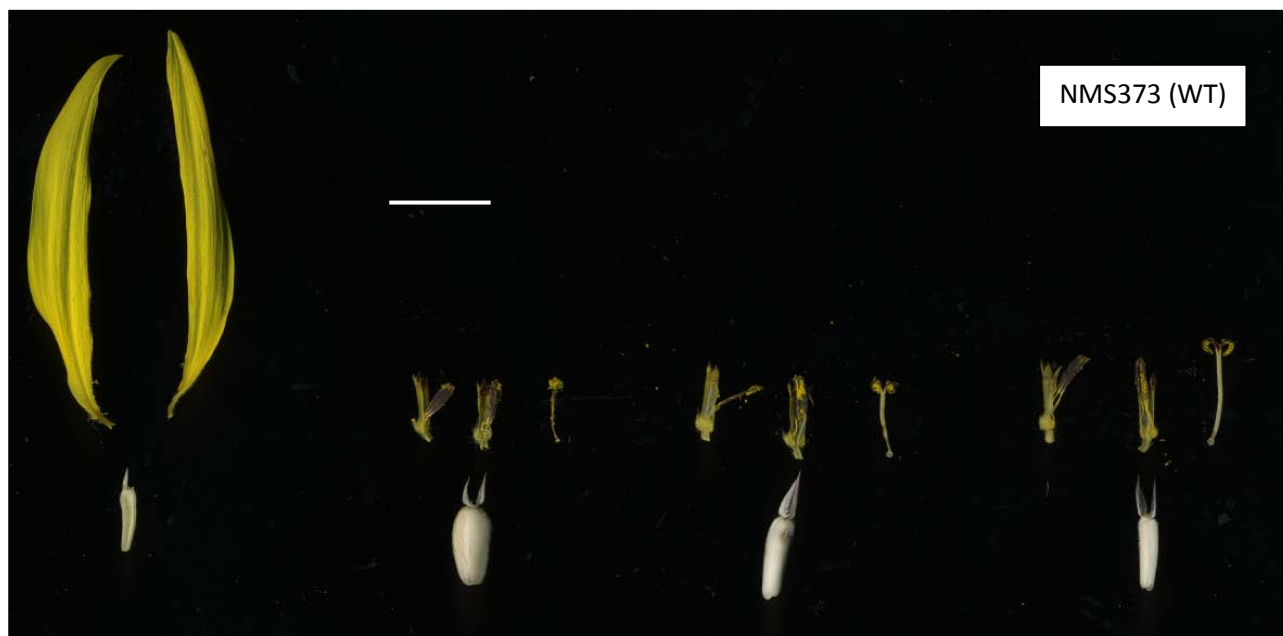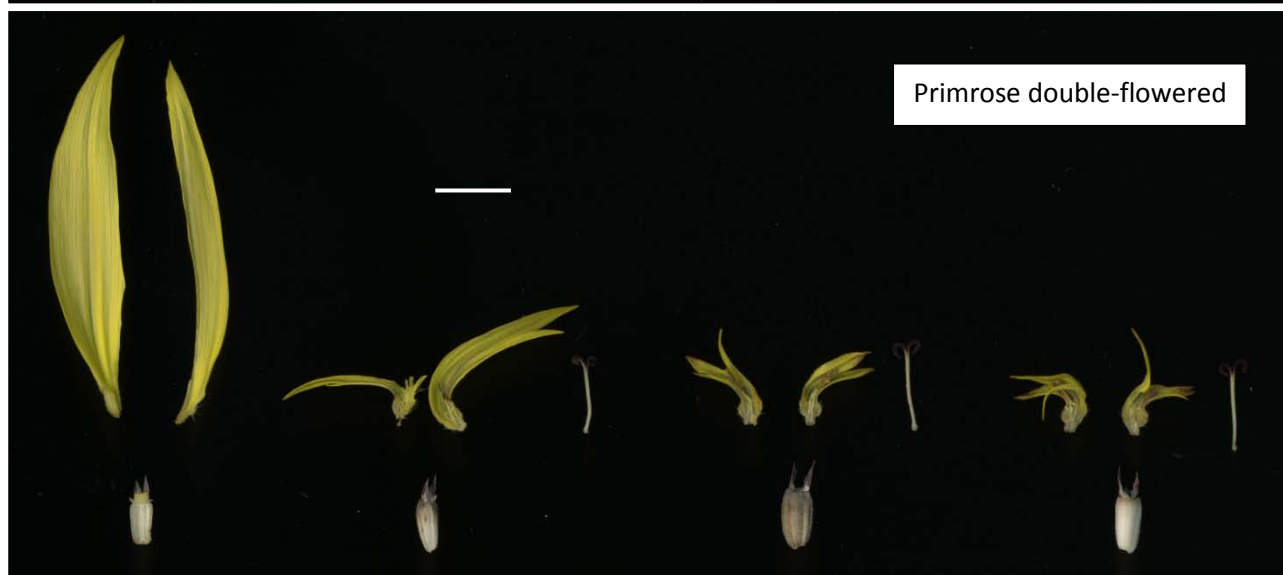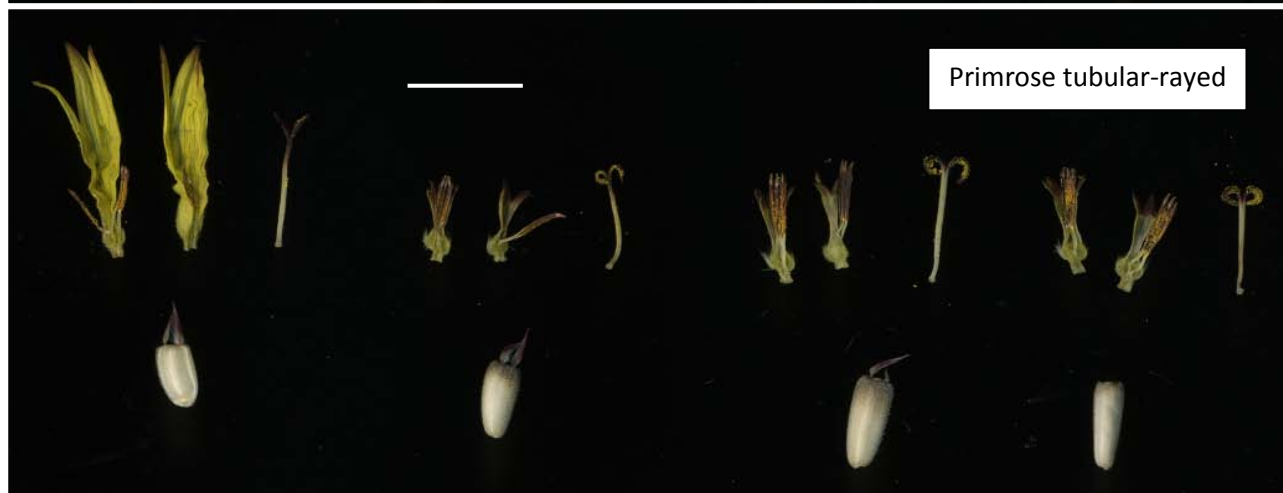

Supplement: Figure S1 — Floret morphology in the WT mutant (Primrose dbl and Primrose tub) lines. Ovaries and stigmas have been removed from the florets, which have been split laterally so that the anthers can be seen. Scale bars (10 mm) are indicated in white. In each panel outer florets are on the left, and the innermost florets on the right. (PDF) [file pgen.1002628.s001.pdf]
