# Supplementary material for: Genetic Analysis of Floral Symmetry in Van Gogh's Sunflowers Reveals Independent Recruitment of CYCLOIDEA Genes in the Asteraceae
Source: PLoS Genet. 2012 Mar 29;8(3):e1002628. doi: 10.1371/journal.pgen.1002628 (PMC3315478; doi:10.1371/journal.pgen.1002628)
Supplement: Table S1 — 108 diverse sunflower lines used in the PCR assay for insertions in Hacyc2c. All lines are available from the USDA (http://www.ars-grin.gov/npgs/index.html) with the exception of SF33 and SF230 available from the French National Institute for Agricultural Research (INRA). (PDF) [file pgen.1002628.s002.pdf]

108 diverse sunflower lines used in the PCR assay for insertions in cyc2c.
All lines are available from the USDA (http://www.ars-grin.gov/npgs/index.html) with the exception of SF33 and SF230 available from the French National Institute for Agricultural Research (INRA)

sunflower core 12 (Mandel et al. 2011):

PI 597368	PI 603989	SF33 (INRA)	PI 476853
PI 599778	PI 617099	SF230 (INRA)	PI 531071
PI 599984	NSL 202284	NSL 208764	PI 531073

96 more lines:

PI 549002	PI 578874	PI 509056	PI 531075
PI 549006	PI 597372	PI 509059	PI 534649
PI 549004	PI 597374	PI 509061	PI 560144
NSL 202854	PI 599762	PI 509063	PI 599753
NSL 202862	PI 599764	PI 509065	PI 543746
NSL 206236	PI 599766	PI 531070	PI 548997
PI 549008	PI 599768	PI 531072	PI 552945
PI 549010	PI 599770	NSL 206235	Ames 22511
PI 549012	PI 599772	NSL 202863	NSL 176426
PI 597365	PI 597376	PI 534653	PI 296289
PI 597367	NSL 202860	PI 534655	PI 650762
PI 597369	PI 599760	PI 534658	PI 369358
PI 549005	PI 597371	PI 509057	PI 531074
PI 549003	PI 597373	PI 509060	PI 534652
PI 549007	PI 599761	PI 509062	PI 597378
NSL 202861	PI 599763	PI 509064	PI 543745
NSL 206235	PI 599765	PI 531069	PI 548996
NSL 208765	PI 599767	PI 531071	PI 548998
PI 549009	NSL 202859	PI 531073	PI 549000
PI 549011	PI 599771	NSL 208765	NSL 166209
PI 549013	PI 597375	Ames 3361	PI 343794
PI 597366	PI 597377	PI 534654	PI 340790
PI 597368	NSL 206234	PI 534656	PI 496264
PI 597370	PI 599759	PI 543744	Ames 19070
