# Supplementary material for: Genetic Analysis of Floral Symmetry in Van Gogh's Sunflowers Reveals Independent Recruitment of CYCLOIDEA Genes in the Asteraceae
Source: PLoS Genet. 2012 Mar 29;8(3):e1002628. doi: 10.1371/journal.pgen.1002628 (PMC3315478; doi:10.1371/journal.pgen.1002628)
Supplement: Table S2 — Primer sequences used throughout this study. (PDF) [file pgen.1002628.s003.pdf]

Supplementary Table 2 - Primer sequences

| | Forward Primer | Reverse Primer |
|---|---|---|
| **PCR and sequencing:** | | |
| *HaCYC2c* CDS (1) | GGTAGAAAATTACACAAAAATGACAGG | TAAGAGACCACCACCCCTCTTA |
| *HaCYC2c* CDS (2) | TAAAGTTTGGAGTGACCGTGTG | TCCCTTGATGGTCTCTAGGAAA |
| *HaCYC2c* CDS (3) | TAGGTTTTGACAAAGCAAGCAA | GGTTTATTTGGACGGATAGATTG |
| *HaCYC2c-dbl* insertion | GCTCATCAACTGGAAACAAAGA | ATTAGCGGCGACCACAGTATTA |
| *HaCYC2c-tub* insertion | CAATCAAACTCTTGGGGTCAAT | CAGTACGTGGATATGGTCATGG |
| *HaCYC2c-3288* insertion | TAATTCACGAGGAGCAGAGTCA | ACATCCAGCACCCTTGAACTAT |
| *HaCYC2b* | ATGCTTTCCTCATCCACCAA | CCTGAAAGCTTGAATCCTTTG |
| *HaCYC2e* (1) | TGGCATTTAACTCCACACAAAA | CCTGAGAGAAATGGGTTGAAGT |
| *HaCYC2e* (2) | TATGTCTTCCCTCCTTCCAATC | CCTGCCTCATCAATGAAACATA |
| **SNP genotyping:** | | |
| *HaCYC2b* | TAATGCCAAGTTTCTGGAAAGC | CTCTTTCTCTTGCCTCTGC |
| *HaCYC2bAnchor** | AAGAGAAGTCATTACTGAAACGTGTGGATGGAAAA | n/a |
| *HaCYC2bSensor#* | GAAGAAAGGGACTCAAAGAGCCAAAGTTA | n/a |
| *HaCYC2c* | TCCACAACCATCAACCCTAC | ATGGGTAAACTACTGACTCCAA |
| *HaCYC2cAnchor** | CCTTCATGATCAAGCAGCTGGTTTTGAC | n/a |
| *HaCYC2cSensor+* | TCTAACTCTTGTGCATTTGAAGGCATAT | n/a |
| *HaCYC2e* | CAAGAGCTAGAGAAAGGACTAA | ATTGTGCTGATAACTATACAGTAA |
| *HaCYC2eAnchor** | AGCTAGACTGGAGTGTTAAATTTGAAGAACAG | n/a |
| *HaCYC2eSensor#* | GTCAACAGGCGCACTCTTGGA | n/a |
| **qRT-PCR** | | |
| *HaCYC2bRT* | CCACTCTTCATCTTCAGCTCTCAC | TGGCTCTTTGAGTCCCTTTCTTCC |
| *HaCYC2cRT* | TTGTTGTATCAGATGAGTGCATCTCCCAA | ATTGCACGGTGACTCTGCTCCTC |
| *HaCYC2eRT* | AGTTGTGTTCCTGGAAACCGACGA | TCTTGCCTCTGCCCTTGACTGATT |
| *ACTIN2RT* | AGGATGAGCAAGGAAATCACGGCT | GTTGGAAGGTGCTGAGTGATGCAA |

* denotes primer was 3' labelled with Fluorescein

\# denotes primer was 5' labelled with Texas Red and a 3' phosphate

\+ denotes primer was 5' labelled with Biodipy630 and a 3' phosphate
